# An Uncommon Case of Incessant Tachycardia-induced Cardiomyopathy in a Child

**DOI:** 10.19102/icrm.2018.090105

**Published:** 2018-01-15

**Authors:** Hermine R. Poghosyan, Arman B. Danoyan, Tatevik B. Hovakimyan, Zara Ed. Kartoyan, Karapet V. Davtyan

**Affiliations:** ^1^Astghik Medical Center, Yerevan, Armenia; ^2^National Medical Research Center for Preventative Medicine, Moscow, Russia

**Keywords:** Ablation, child, incessant supraventricular tachycardia, uncommon concealed slow accessory pathway

## Abstract

The case of a pediatric patient with a history of incessant narrow complex tachycardia is presented. The patient underwent successful catheter ablation for an uncommon concealed slow accessory pathway. The mechanism and ablation location are discussed.

## Case presentation

**Figure 1:**
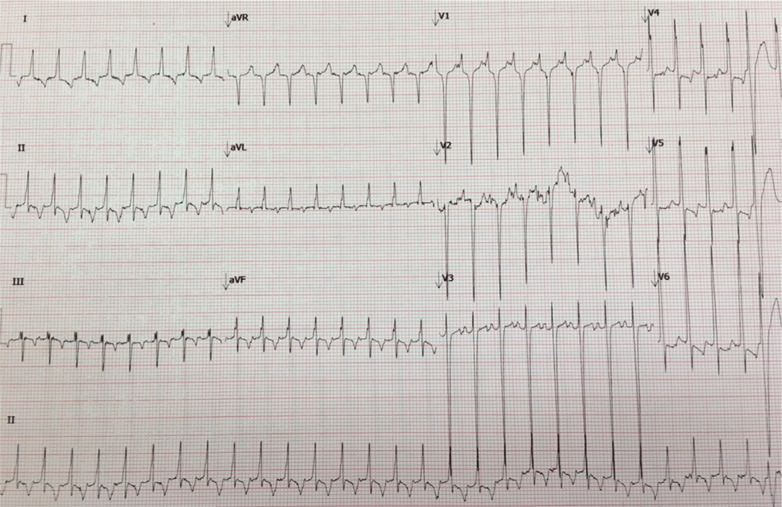
Supraventricular tachycardia (SVT) with a long RP interval was recorded on electrocardiogram (ECG). Positive P-waves are seen in V1.

A nine-year-old girl was admitted to our clinic having presented with palpitation, dyspnea, and heart failure (New York Heart Association functional classification class II). A 12-lead resting electrocardiogram (ECG) revealed regular narrow complex tachycardia **(Figure 1)**. Echocardiography revealed reduced left ventricular systolic function (ejection fraction was 35%) and normal biatrial diameter. Tachycardia was incessant, lasting more than 50% of monitoring time prior to drug administration. Intravenous adenosine and β-blocker medication were ineffective. The patient was referred for electrophysiology (EP) study and catheter ablation. The procedure was performed with the patient under sedation with intubation. A steerable decapolar catheter (Abbott Laboratories, Chicago, IL, USA) was inserted into the coronary sinus via the subclavian vein, a quadripolar catheter (Abbott Laboratories, Chicago, IL, USA) was positioned in the right ventricle, and an ablation catheter (Marinr^®^ MC; Medtronic, Minneapolis, MN, USA) was placed in the His position via the right femoral vein during the EP study. An AXIOM Sensis XP system (Siemens AG, Munich, Germany) was also used during the procedure.

**Figure 2:**
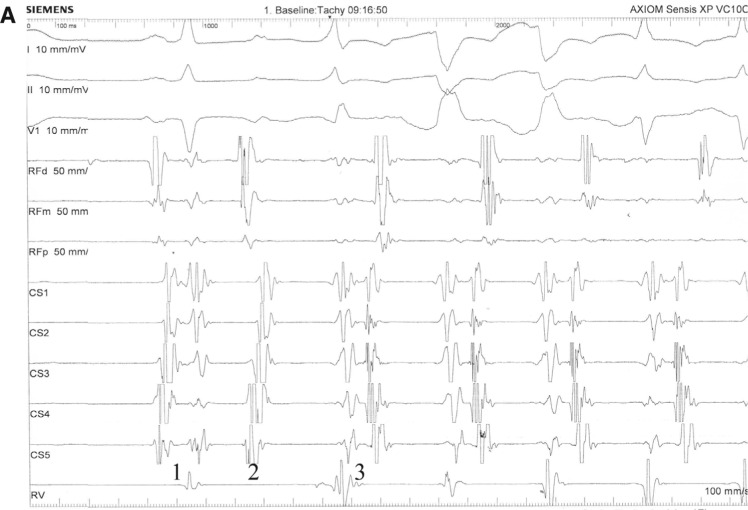

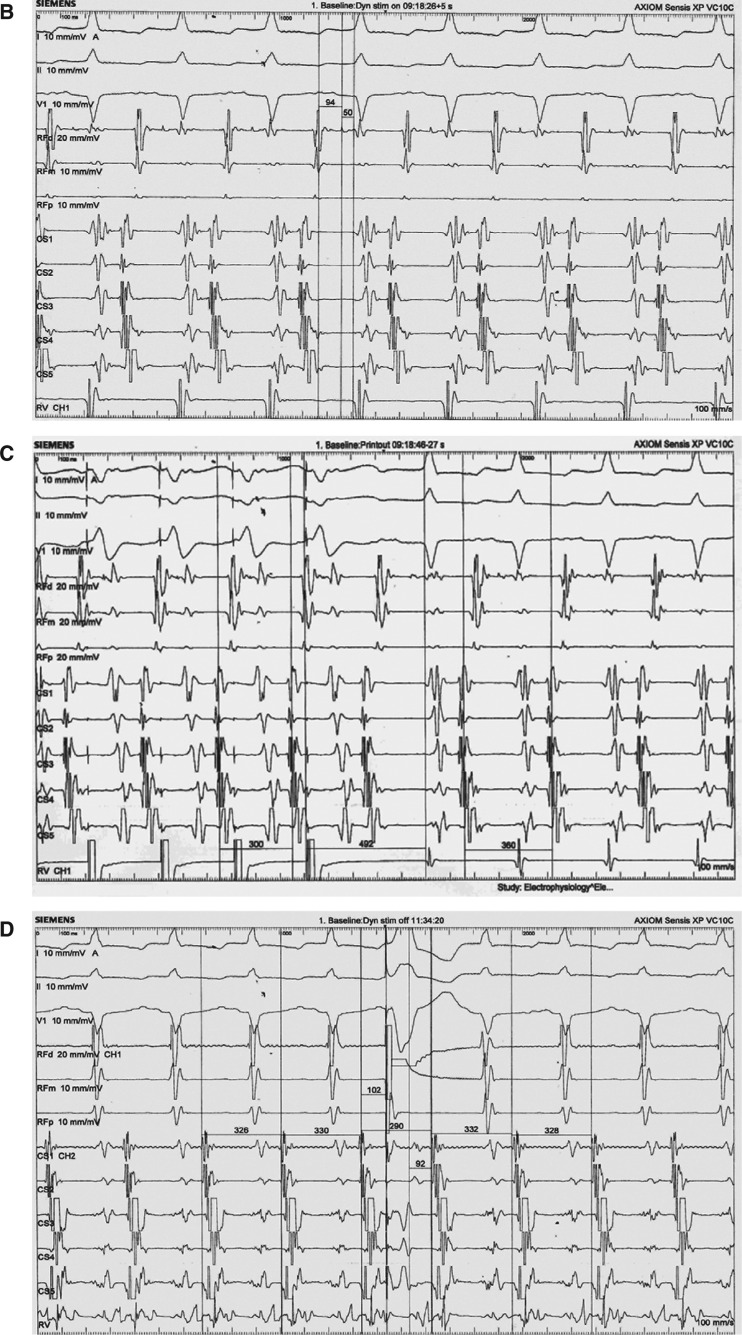

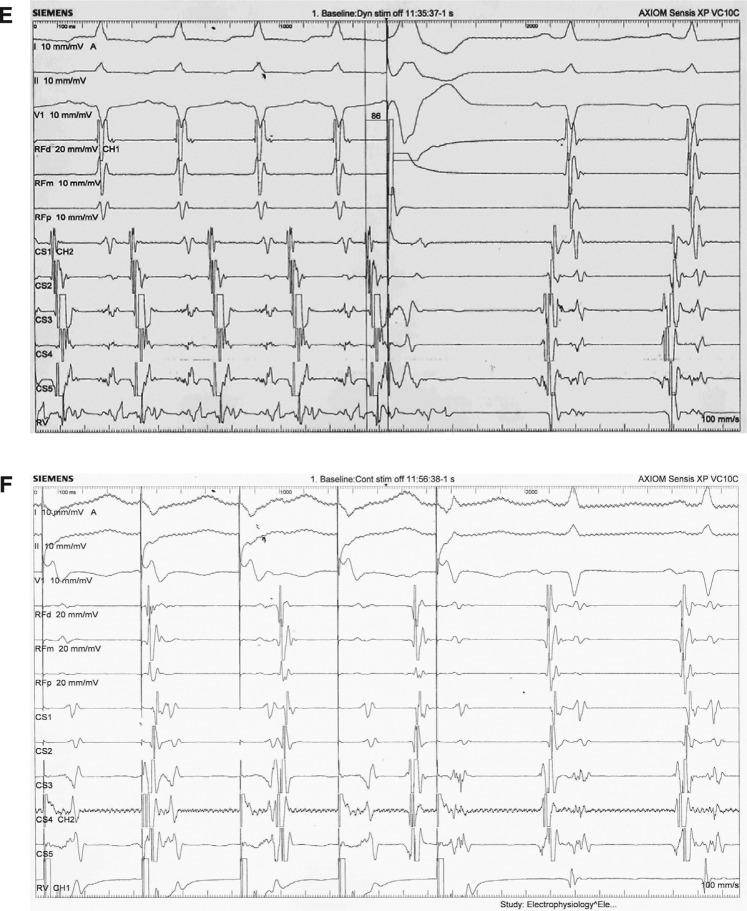
**A:** An RF catheter is positioned in the tricuspid annulus. The first beat (1) was sinus and conducted to the ventricle through the AV node. The second beat (2) was a premature atrial conduction conducted to the ventricles by the AV node (long AV interval), which initiated (3) SVT with a cycle length (CL) of 360 ms and a constant ventriculoatrial (VA) interval of 104 ms. Aberrancy in the first tachycardic beats was recorded. **B:** His potential during tachycardia. The atrium to His interval was 94 ms; the His to ventricle interval was 50 ms. **C:** Overdrive pacing from the right ventricle was performed. Entrainment was confirmed by measurement of the AA interval during pacing (300 ms) and tachycardia (360 ms), which was also the tachycardia CL. VAV response and 492-ms PPI are illustrated. PPI-TCL = 132 ms. The earliest atrial activations were detected in CS2, positioned in the left posteroinferior wall. **D:** A PVC placed at the time of His bundle refractoriness advanced the atrium without changing the atrial activation sequence and reset the tachycardia, which was diagnostic for a retrograde pathway that participates in tachycardia. **E:** The ventricular pacing given earlier terminated tachycardia without advancing to the atrium. **F:** Retrograde ventriculoatrial dissociation was recorded during right ventricular pacing after ablation.

Narrow complex tachycardia with a cycle length (CL) of 360 ms was ongoing. The earliest atrial activations were recorded in the left posterolateral wall (ie, at 4 o’clock according to the clinical standard nomenclature by Cosio) with the shortest ventriculoatrial (VA) interval equal to 104 ms. Few sinus complexes were recorded with concentric ventricular activation **(Figure 2A)**. The atrium to His (AH) and His to ventricle intervals were 94 ms and 50 ms, respectively **(Figure 2B)**. The post-pacing interval was 492 ms with a ventricle-atrium-ventricle (VAV) response during overdrive pacing and entrainment of tachycardia from the right ventricle **(Figure 2C)**. Ventricular pacing within 40 ms of the His potential advanced the atrium and the atrial activation sequence to the same as that seen during tachycardia **(Figure 2D)**. The ventricular pacing given earlier terminated the tachycardia without advancing to the atrium **(Figure 2E)**. The VA interval was not decremental, although the tachycardia CL fluctuated from 310 ms to 360 ms. Because of the incessant tachycardia, we could not pace the atrium in sinus rhythm; however, the short attempts showed that the AH intervals during the sinus complexes and the atrial pacing in tachycardia CL were similar. Orthodromic atrioventricular reentrant tachycardia (AVRT) with concealed slow conductive accessory pathway (AP) was diagnosed.

**Figure 3:**
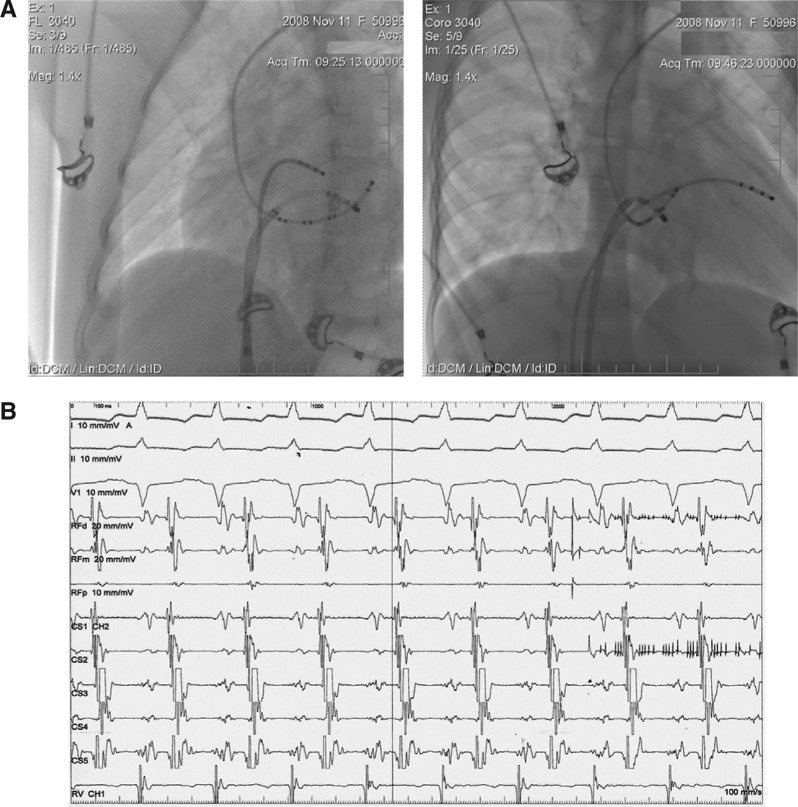
**A**: Fluoroscopic imaging of catheter positions during the successful ablation (right: RAO, left: LAO). The decapolar catheter was in the coronary sinus. The Marinrs MC ablation catheter (Medtronic, Minneapolis, MN, USA) was in the mitral groove. The quadripolar catheter was in the right ventricle. **B:** Local activation at the successful ablation site. No VA fusion was present at the ablation site.

Trans-septal puncture was performed using a Brockenbrough curved needle (Abbott Laboratories, Chicago, IL, USA). Radiofrequency ablation was performed in the posterolateral wall of the mitral annulus. Supraventricular tachycardia (SVT) terminated at the fifth second of ablation and did not recur. Stable VA dissociation was recorded **(Figure 2D)**. Tachycardia was no longer inducible after a 30-minute waiting period. No other coarrhythmias were included. The procedure was completed without complication. Fluoroscopy time was 10 minutes **(Figure 3A)**. Local activation at the successful ablation site is shown in **Figure 3B**.

No SVT recurrence was detected during the three-month follow-up period, and left ventricular ejection fraction was 58% soon after the procedure.

## Discussion

Limited literature is available on the association between incessant AVRT and tachycardia-induced cardiomyopathy in pediatric patients. Generally, the incessant nature of tachycardia favors permanent junctional reciprocating tachycardia (PJRT).^1^ The AP in PJRT usually has retrograde and anterograde decremental conduction properties and is typically identified in the posteroseptal location.^1^ Critelli et al. described the anatomical substrate of PJRT as an AP with a tortuous course through the AV annulus fenestration, which probably causes its decremental properties.^2^ The surface ECG criteria for PJRT are well reported and include narrow QRS tachycardia with negative P-waves in inferior leads, a PR interval shorter than the RP interval, an atrioventricular (AV) ratio of 1:1, no evidence of a delta wave during sinus rhythm, and no episode of functional AV block during tachycardia.^3^ The patient’s ECG met all these criteria, but the P-waves were considered positive in inferior leads. AVRT, atrial tachycardia, and atypical AV nodal reentrant tachycardia (AVNRT) must be excluded in such cases **(Figure 1)**.

A previous study reported that only 2.9% (34 patients) of 1,163 consecutive individuals with Wolff-Parkinson-White syndrome had VA conduction times greater than 80 ms.^4^ Only one of these patients was younger than 18 years.^4^ The authors reported long ablation times while treating this type of pathway.

The difference between the present case and patients with PJRT is the retrograde circuit, which is capable of slow retrograde conduction over an AP without decremental properties and is located in the posterolateral zone. In our case, procedural time was also longer than usual (ie, 100 minutes).

Notably, we observed a VAV response during ventricular overdrive pacing **(Figure 2C)**, although it is known that a pseudo-ventricle-atrium-atrium-ventricle (pseudoVAAV) response may occur in such a situation.^5^ As reported previously, a pseudo-VAAV response occurs when retrograde conduction is slow either in an atypical AVNRT or a slow AP with retrograde conduction.^5^ A postpacing interval-tachycardia CL (PPI-TCL) > 115 ms is usually consistent with AVNRT, while a PPI-TCL difference < 115 ms is consistent with AVRT.^6^ Nevertheless, a PPI-TCL > 110 ms can occur with AVRT circuited by a left-sided AP, because the right ventricular pacing site is far from such a circuit. During a long RP interval SVT, a corrected PPI-TCL > 110 ms should also prompt the consideration of orthodromic AVRT employing an AP with delayed retrograde conduction.^7^ The most common maneuver is to deliver a His-synchronous premature ventricular contraction (PVC) on time or within 30 ms of the His potential. Unfortunately, figures of His refractory pacing, in which the His potential was recorded, are not presentable in the current case due to noise. In this case, atrial activation is advanced without a change in the atrial activation sequence. The fact that a His refractory PVC can affect atrial timing indicates that an AP is present. As the atrial activation sequence is unaltered, we conclude, though not with certainty, that the AP is participating in the SVT circuit, establishing a diagnosis of AVRT **(Figure 2D)**. The earlier PVC terminates tachycardia without conduction to the atrium. This indicates that the AP is present and is part of the circuit **(Figure 2E)**.

The AH interval also has an important diagnostic role in the EP study. During PJRT, this interval is a true interval, similar to the AH interval, when pacing at the tachycardia CL (difference of < 20 ms).^8^ EP criteria of long RP tachycardia involving concealed nodofascicular APs includes a shorter AH interval during tachycardia compared with during sinus rhythm.^8^ In our case, because of the incessant tachycardia, we could not pace the atrium in sinus rhythm. However, the AH intervals during SVT and atrial pacing were similar to that during sinus rhythm. A short AH interval is also reportedly an important factor in the development of incessant tachycardia.^9^

Previous studies have shown that local VA at the successful ablation site is usually 25 ms to 50 ms and that a VA interval of less than 50 ms is an independent predictor for successful AP ablation.^10^ Lin et al. described the EP characteristics of an AP with a long VA interval (arbitrarily defined as ≥ 50 ms with an absence of continuous electrical activity) and no retrograde decremental property.^11^ Fifteen patients with the aforementioned characteristics were compared with 171 study participants with normal VA conduction; the mean VA conduction time was 77 ms ± 24 ms. Notably, the experimental group included significantly older patients with longer retrograde AP block CLs and retrograde AP effective refractory periods. In these patients, adenosine and verapamil were ineffective for terminating tachycardia. There was also a positive correlation between AP VA interval and patient age. In this current case, we observed this rare pathway in a pediatric patient, and there was no VA fusion at the successful ablation site.

## Conclusions

Slow retrograde conducted AP may be located in an unusual left posterolateral site. In this case, it induced incessant tachycardia with cardiomyopathy in a child. The EP features of this pathway are an absence of AV fusion in the ablation site and long PPI during entrainment from the right ventricle.
